# Design of YΦ motif peptides to promote 20S proteasome mediated degradation of tau

**DOI:** 10.1002/alz.095619

**Published:** 2025-01-09

**Authors:** Bryan D. Ryder, Nicholas L. Yan, Jason E. Gestwicki, ALBORADA Drug Discovery Team

**Affiliations:** ^1^ University of California, San Francisco, San Francisco, CA USA

## Abstract

**Background:**

An optimized 6 amino acid peptide (NLSYYT; herein YΦ) derived from the C‐terminus of h19S proteasome activator Rpt5 has been shown to activate the 20S proteasome and promote tau degradation. Further analysis of this peptide has identified the highly conserved leucine in position 5 (P5) as a key part of the 20S activation mechanism to drive degradation of tau monomers in the absence of proteasome activator complexes.

**Method:**

Recombinant peptides were used to identify key amino acids required for binding and activating the h20S proteasome. Degradation activity from YΦ‐mediated activation was measured with small peptide markers. Using archaeal activator complex aPA26 with YΦ and P5 mutants cloned onto its tails, P5 was shown to use hydrophobic interactions to stabilize YΦ interactions with the h20S α‐subunit ring. Finally, YΦ activation of the h20S proteasome increased tau degradation *in vitro*, in cell lysates, and in cells.

**Result:**

*In vitro* tau degradation experiments show that simultaneous binding of linked YΦ peptides is sufficient to promote degradation of tau monomer in an ATP‐independent and ubiquitin‐independent system. Mutations to the P5 leucine result in a 3‐fold reduction in h20S proteasome activity for aromatic substitutions, and complete loss of activity for all other natural amino acid substitutions. However, P5 substitutions do not have as dramatic of an effect on binding, demonstrating that the role of P5 leucine uncouples activity and binding, unlike the other amino acids. In the context of aPA26, YΦ mediated activation clears 80% of native tau monomer over the course of 30 minutes, with similar results observed in degrading tau derived from cell culture.

**Conclusion:**

Structure‐activity relationship data of YΦ activation of the 20S proteasome provides insight into future designs of proteasome activators. With an improved understanding of the role of P5 leucine, a more refined interaction network can be considered when designing h20S proteasome activators for clearing disease‐associated tau in cells.

